# Climbing the longevity pyramid: overview of evidence-driven healthcare prevention strategies for human longevity

**DOI:** 10.3389/fragi.2024.1495029

**Published:** 2024-11-26

**Authors:** Anđela Martinović, Matilde Mantovani, Natalia Trpchevska, Eva Novak, Nikolay B. Milev, Leonie Bode, Collin Y. Ewald, Evelyne Bischof, Tobias Reichmuth, Rebecca Lapides, Alexander Navarini, Babak Saravi, Elisabeth Roider

**Affiliations:** ^1^ Maximon AG, Zug, Switzerland; ^2^ Department of Food Environmental and Nutritional Sciences (DeFENS), University of Milan, Milan, Italy; ^3^ Avea Life, Zug, Switzerland; ^4^ AYUN, Zug, Switzerland; ^5^ Laboratory of Extracellular Matrix Regeneration, Institute of Translational Medicine, Department of Health Sciences and Technology, ETH Zürich, Zürich, Switzerland; ^6^ Shanghai University of Medicine and Health Sciences, Shanghai, China; ^7^ Sheba Longevity Center, Sheba Medical Center Tel Aviv, Ramat Gan, Israel; ^8^ The Robert Larner, M.D., College of Medicine at the University of Vermont, Burlington, VT, United States; ^9^ Department of Dermatology, University Hospital Basel, Basel, Switzerland; ^10^ Department of Orthopedics and Trauma Surgery, Medical Center - University of Freiburg, Faculty of Medicine, University of Freiburg, Freiburg, Germany; ^11^ Department of Dermatology, University Hospital of Basel, Basel, Switzerland; ^12^ Cutaneous Biology Research Center, Massachusetts General Hospital, Harvard Medical School, Charlestown, MA, United States

**Keywords:** longevity, healthcare, aging, lifestyle, preventive medicine, personalized medicine

## Abstract

Longevity medicine is an emerging and iterative healthcare discipline focusing on early detection, preventive measures, and personalized approaches that aim to extend healthy lifespan and promote healthy aging. This comprehensive review introduces the innovative concept of the “*Longevity Pyramid*.” This conceptual framework delineates progressive intervention levels, providing a structured approach to understanding the diverse strategies available in longevity medicine. At the base of the Longevity Pyramid lies the level of prevention, emphasizing early detection strategies and advanced diagnostics or timely identification of potential health issues. Moving upwards, the next step involves lifestyle modifications, health-promoting behaviors, and proactive measures to delay the onset of age-related conditions. The Longevity Pyramid further explores the vast range of personalized interventions, highlighting the importance of tailoring medical approaches based on genetic predispositions, lifestyle factors, and unique health profiles, thereby optimizing interventions for maximal efficacy. These interventions aim to extend lifespan and reduce the impact and severity of age-related conditions, ensuring that additional years are characterized by vitality and wellbeing. By outlining these progressive levels of intervention, this review offers valuable insights into the evolving field of longevity medicine. This structured framework guides researchers and practitioners toward a nuanced strategic approach to advancing the science and practice of healthy aging.

## 1 Introduction

The primary focus of medicine in the late 19^th^ and early 20^th^ centuries was the management of communicable diseases (Sakai and Morimoto, 2022). While significant progress has been made in combating certain communicable diseases on a global scale, other infectious diseases continue to pose considerable challenges in various parts of the world. Today’s healthcare systems confront a different landscape: the prevalence of chronic diseases, which often develop over extended periods, with the most critical being the *“top four”*: cardiovascular diseases, cancer, chronic respiratory diseases, and diabetes (Booth et al., 2000; Budreviciute et al., 2020). Modern medicine has adapted various strategies in response to this shift, yet there is a tendency for chronic disease management to mirror approaches historically used for infectious diseases (Budreviciute et al., 2020). This has sometimes led to interventions being applied in the later stages of chronic diseases as symptom management becomes the predominant focus rather than early prevention. As a result, the healthcare stance today is often reactive, rather than proactive–addressing illness once it has already manifested. Without the adoption of new medical and wellness paradigms, the world is set to face an unsustainable burden of chronic diseases, which is already taking a substantial social and economic toll (Gmeinder et al., 2017). To mitigate the age gradient in comorbidities, a health system focused on prevention rather than intervention is imperative. A shift in mindset is therefore needed, necessitating a transition toward long-term prevention strategies that align more appropriately with the gradual progression inherent to chronic diseases.

The emerging field of longevity medicine represents a rapidly evolving multidisciplinary healthcare domain. It is dedicated to comprehending and extending the span of a healthy human life–a concept known as *“healthspan”* – rather than simply extending life in general (Garmany et al., 2021). Longevity medicine incorporates a range of strategies, including lifestyle adjustments, preventative healthcare measures, pharmaceutical interventions, and state-of-the-art medical technologies, all aimed at promoting healthy aging, delaying the onset of age-related diseases, and enhancing the quality of life during the later stages of life (Bischof et al., 2021; Ruckstuhl et al., 2023). This field integrates principles from biology, genetics, nutrition, exercise physiology, and medical science to address the intricate and multifaceted aspects of aging, aiming to enable individuals to live longer and healthier lives.

**FIGURE 1:**
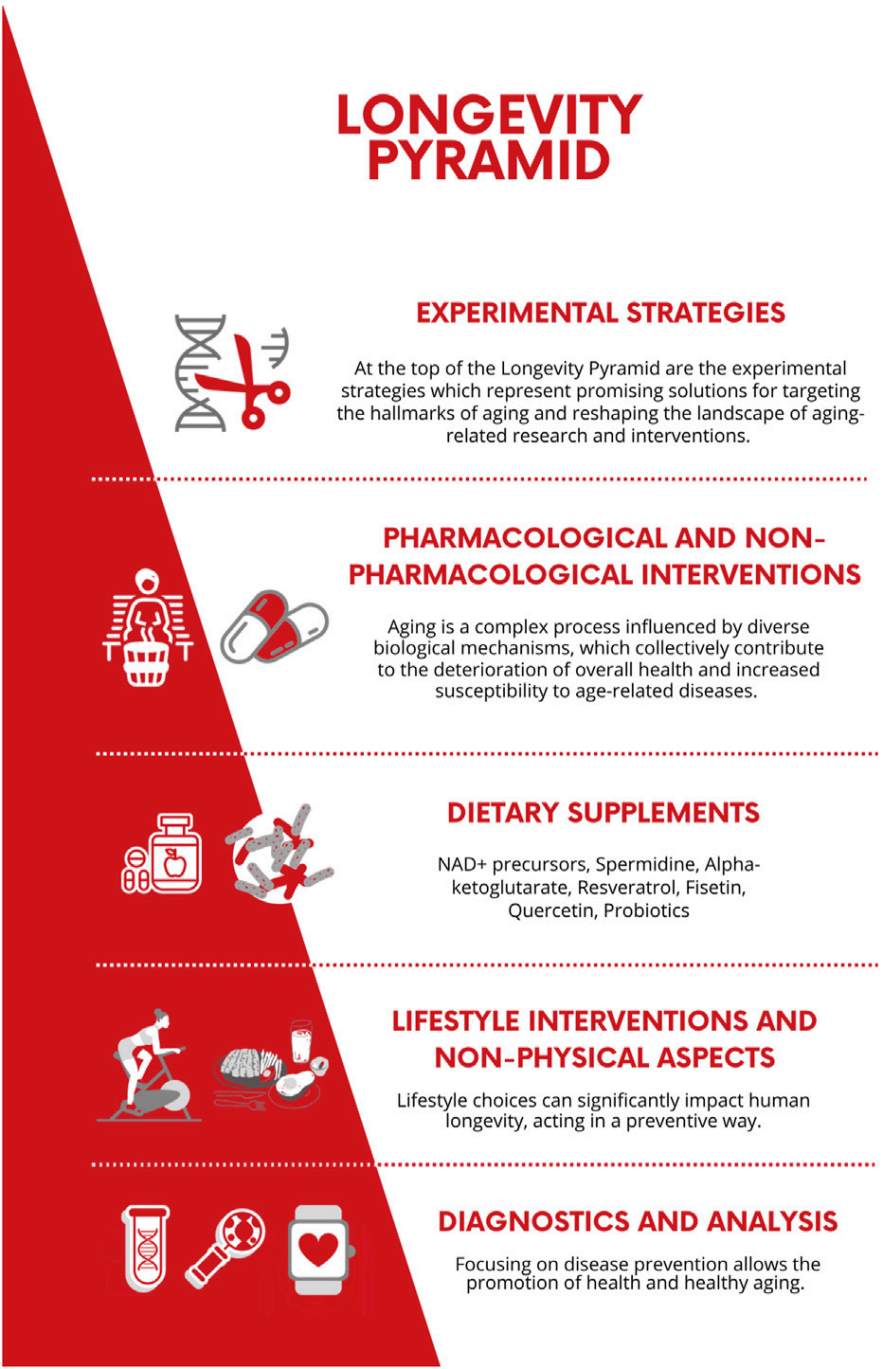
Schematic representation of the Longevity Pyramid, illustrating healthcare prevention strategies to promote human lifespan. The layers include 1) Diagnostic and Analysis; 2) Lifestyle Interventions and Non-Physical Aspects; 3) Dietary Supplements; 4) Pharmacological and Non-pharmacological Interventions, and 5) Experimental Strategies.

The present narrative review aims to provide insight into the “longevity pyramid” concept, a structure that effectively describes the various levels of longevity medicine interventions (Figure 1). These levels start with preventive measures and diagnostics analysis, progressing through a series of intermediate stages and culminating in laboratory experimental strategies, representing the scientific trends, and setting the current and future challenges for developing new therapeutic targets.

In conducting this review, we focused on synthesizing relevant literature by searching major databases, including PubMed, Web of Science, Embase, and Google Scholar. Additionally, references from selected studies of interest were reviewed and included where relevant. Our approach was deliberately narrative rather than systematic, given the broad scope and complexity of longevity medicine, which spans diverse fields such as genetics, lifestyle interventions, and medical technologies. A systematic review, requiring narrow research questions and rigid inclusion/exclusion criteria, would not have captured the complexity and evolving nature of this multidisciplinary field. We used search terms such as ‘longevity medicine,’ ‘healthy aging,’ ‘preventive healthcare,’ ‘biomarkers of aging,’ and ‘experimental aging interventions,’ conducting searches from inception to August 2024 to ensure a comprehensive overview of the current scientific landscape.

## 2 Diagnostics and analysis

Longevity medicine is an iterative healthcare model based on early detection, prevention, and deep personalization. Focusing on disease prevention allows the promotion of health and healthy aging. Biological age, a measure of aging based on various biomarkers and differing from chronological age, which simply counts the years since birth, presents a complex challenge in accurate prediction. Currently, no single test can precisely assess biological age, as it is influenced by the choice of aging biomarkers, the standards applied, and the statistical method used, including approaches like multiple linear regression (MLR), principal component analysis (PCA), Klemera and Doubal’s method (KDM), and recent deep learning methods (Li X. et al., 2023). Despite significant advancements in this field in recent years, numerous technical challenges remain. It appears more plausible that in order to get a more accurate picture, the integration of data from different sources will be necessary (Chen et al., 2023). This may include genetic and epigenetic testing, blood biomarkers, as well as physiological testing, and continuous biometric measurements through wearable devices.

With the advancements in aging research, the identification and evaluation of biomarkers of aging have become increasingly crucial in promoting precise longevity interventions (Moqri et al., 2023). Many effective tests can indicate whether progress is being made in the right direction. For instance, blood biomarkers can be used to reveal disease risk, while physiological data can track improvements in fitness which are associated with longevity (McGreevy et al., 2023). Having a well-defined testing protocol is essential to identify an individual’s risk factors and opportunities for intervention. In the context of longevity medicine, testing is performed iteratively: the initial measurement sets the baseline, and subsequent regular tests help to assess the efficacy and guide the process.

### 2.1 Hematologic biomarkers

Hematologic biomarkers, which include a variety of proteins, metabolites, and genetic markers, have become valuable tools for assessing overall health, early disease detection, risk assessment, tracking disease progression, and predicting mortality. This comprehensive and minimally invasive approach to diagnostics is not only beneficial for patients but also enables more effective and efficient healthcare delivery, ultimately contributing to improved patient outcomes and the advancement of medical science (Ubaida-Mohien et al., 2023). Common blood tests conducted by healthcare professionals to assess overall health include measuring blood levels of glucose, cholesterol, thyroid hormones, and C-reactive protein (CRP) and assessing mineral and vitamin levels. Other blood biomarkers are also employed to detect various diseases, including organ dysfunctions and hormone imbalances (Table 1).

**TABLE 1:** Commonly performed blood tests to assess overall health or diagnose specific medical condition (s).

Blood test	Biomarker/s	Disorder/Disease
Complete Blood Count (CBC)	Red blood cells, white blood cells, platelets	Anaemia, leukaemia
Glucose Test	Glucose, HbA1c	Diabetes
Lipid Panel Test	LDL, HDL, triglycerides	Heart-related diseases
Liver Function Test	ALT, AST, ALP, albumin, bilirubin, GGT, LDH, PT	Liver damage, hepatitis, cirrhosis
Kidney Function Test	Creatinine, Cystatin C, BUN	Kidney damage
Thyroid Tests	TSH, Thyroglobulin, T3, T4	Hypothyroidism, Hyperthyroidism
C-reactive Protein (CRP)	CRP	Infections, injuries, autoimmune disorders, lung diseases
Mineral Levels	Iron, Zinc, Potassium, Magnesium	Nutritional deficits
Vitamin Levels	Vitamin D, B9, B12, C	Nutritional deficits
Alzheimer’s Disease (AD) test	Aβ40/Aβ42 ratio, p-tau, NfL	Early AD diagnosis

Abbreviations: HbA1c - glycated haemoglobin; LDL, low-density lipoprotein; HDL, high-density lipoprotein; ALT, alanine transaminase; AST, aspartate aminotransferase; ALP: alkaline phosphatase; GGT, gamma-glutamyl transferase; LDH, lactate dehydrogenase; PT: prothrombin time; BUN: blood urea nitrogen; TSH: thyroid stimulating hormone.

To date, no specific hematologic biomarkers have yet been widely adopted for routine clinical diagnosis of neurodegenerative diseases (Angioni et al., 2022). However, some promising blood-based marker candidates are under investigation for early detection of abnormal amyloid status in cognitively impaired and cognitively unimpaired subjects (Table 1).

### 2.2 Genetic and epigenetic tests

In modern medicine, elucidating genetic and epigenetic information is becoming increasingly essential in a new era of personalized healthcare and diagnostics. In the context of longevity, genetic and epigenetic testing, through the analysis of an individual’s genetic code and epigenetic modifications, have become indispensable tools that provide profound insights into an individual’s susceptibility to diseases and their response to therapeutic interventions (Passarino et al., 2016). Genetic tests are employed to identify genetic mutations with the potential to provide insights into inherited traits, the risk of developing certain diseases, the potential for adverse drug reactions, and the impact of a patient’s capacity to assimilate and/or utilize specific nutrients. Further, the outcomes of these genetic tests have been demonstrated to facilitate behavioral changes, with the testing serving as a catalyst for informed health decisions and lifestyle modifications (Horne et al., 2018).

Meanwhile, epigenetic testing screens the modifications that regulate gene expression without altering the underlying DNA sequence. For instance, altered DNA methylation has been linked to the initiation and progression of tumors (Beltrán-García et al., 2019). Moreover, this epigenetic marker can predict the biological age in mammals, serving as a potential indicator of life expectancy (Hannum et al., 2013; Johansson et al., 2013; Rutledge et al., 2022).

Genetic and epigenetic testing is important in disease diagnosis and predicting an individual’s susceptibility to various conditions, ranging from hereditary disorders to complex multifactorial diseases. These tests can reveal vital information for preventive healthcare, allowing for early interventions that substantially improve patient outcomes. Furthermore, genetic, and epigenetic data can guide treatment decisions, enabling a more tailored and effective approach to patient care.

### 2.3 Wearable healthcare technologies

Wearable devices are innovative and non-invasive technologies that are integrated into daily life and offer unprecedented access to real-time health data, enabling individuals to actively to actively engage and participate in their own health monitoring and improvement (Smuck et al., 2021). Examples of wearable healthcare monitoring devices include health watches, fitness trackers, smart rings, and glucose monitors, which can track vital signs such as heart rate, blood pressure, sleep quality, and body temperature (Smith et al., 2023). These devices provide healthcare professionals with valuable patient information in healthcare settings, facilitating timelier and data-driven decision-making. One of the key strengths of wearable technologies is their ability to continuously monitor vital signs, detect anomalies, and track health parameters. This constant data stream can lead to early detection of health issues and the ability to intervene proactively (Ajami and Teimouri, 2015). Moreover, wearable devices have the potential to personalize healthcare, as the collected data can monitor an individual’s adaptation to treatments and lifestyle changes, leading to more personalized interventions.

### 2.4 Physiological measurements

The importance of physiological measurement and testing in healthcare lies in its ability to deliver precise and actionable insights into an individual’s health status. Through a vast spectrum of techniques, physiological and fitness testing allows healthcare practitioners to assess organ function and detect abnormalities (Table 2). These diagnostic applications can be used, for example, to assess cardiac physiology, respiratory physiology, muscle strength, and muscle endurance (Duren et al., 2008; Pate et al., 2012; Ojeda et al., 2020; Naqvi and Sherman, 2023). Physiological measurements are crucial components in the early detection, diagnosis, and management of various medical conditions. Tailoring interventions to an individual’s unique physiological profile fosters a patient-centric approach that leads to more effective treatments and improved outcomes.

**TABLE 2:** Commonly performed physiological and fitness tests. They provide a comprehensive understanding of overall health, aiding in the assessment of an individual’s treatment journey.

Physiological test	Body measurement	Methods
Body Composition	Weight, stature, abdominal circumference, skinfold measurements	BIA, BMI, X-ray tomography
Cardiovascular Endurance	Hearth and Lung efficiency in supplying oxygen	Treadmill run tests, VO2 max tests, stationary bike
Muscle Strength	Muscle force	Sit-up, push-up, core strength, grip strength and stability tests
Muscle Endurance	Muscle contraction and release	Sit-up, push-up, core strength and stability tests
Flexibility	Shoulder, lower back, hamstring, trunk	Shoulder flexibility, sit-and-reach tests, trunk lift

Abbreviations: BIA, Bioelectrical Impedance Analysis; BMI, Body Mass Index; MRI, Magnetic Resonance Imaging.

## 3 Lifestyle interventions and non-physical aspects

Promoting longevity involves adopting a healthy lifestyle encompassing various aspects of daily routine. Lifestyle choices can significantly impact human longevity, acting in a preventive way, thus delaying the onset and/or progress of age-related and mainly non-communicable diseases (Fernández-Ballesteros et al., 2021). In this section, we will dive deeper into exercise, physical activity, and dietary interventions, as critical lifestyle interventions impacting one’s longevity.

### 3.1 Exercise and physical activity

Exercise, often regarded as a “*longevity drug,*” is undoubtedly a promising lifestyle intervention that positively affects both health span and lifespan, and it is seen as the foremost lifestyle intervention for promoting longevity (Vina et al., 2012). Exercise, unlike other interventions such as caloric restriction, which has been associated with adverse outcomes like lean mass loss and cardiovascular maladaptation (Schafer et al., 2019), should be considered a primary strategy for promoting longevity. The longevity benefits of exercises are attributed mainly to the attenuation of aging phenotypes or by delaying the process of aging by decreasing the hallmarks of aging and age-associated inflammation (Qiu et al., 2023).

The positive effects of regular exercise are well-established for the prevention, treatment, and management of chronic disease, significantly influencing longevity (Nocon et al., 2008; Qiu et al., 2023). Studies highlight that maintaining a minimum level of exercise enhances cardiorespiratory fitness, muscle function, flexibility, balance, and mental health (Carapeto and Aguayo-Mazzucato, 2021; D’Onofrio et al., 2023). Notably, the benefits of exercise on longevity are observed even in individuals with genetically determined longevity, such as centenarians, where the decline in lung function and sarcopenia could be counteracted by exercise programs that increase their physical capacity and health span (Venturelli et al., 2012).

**FIGURE 2:**
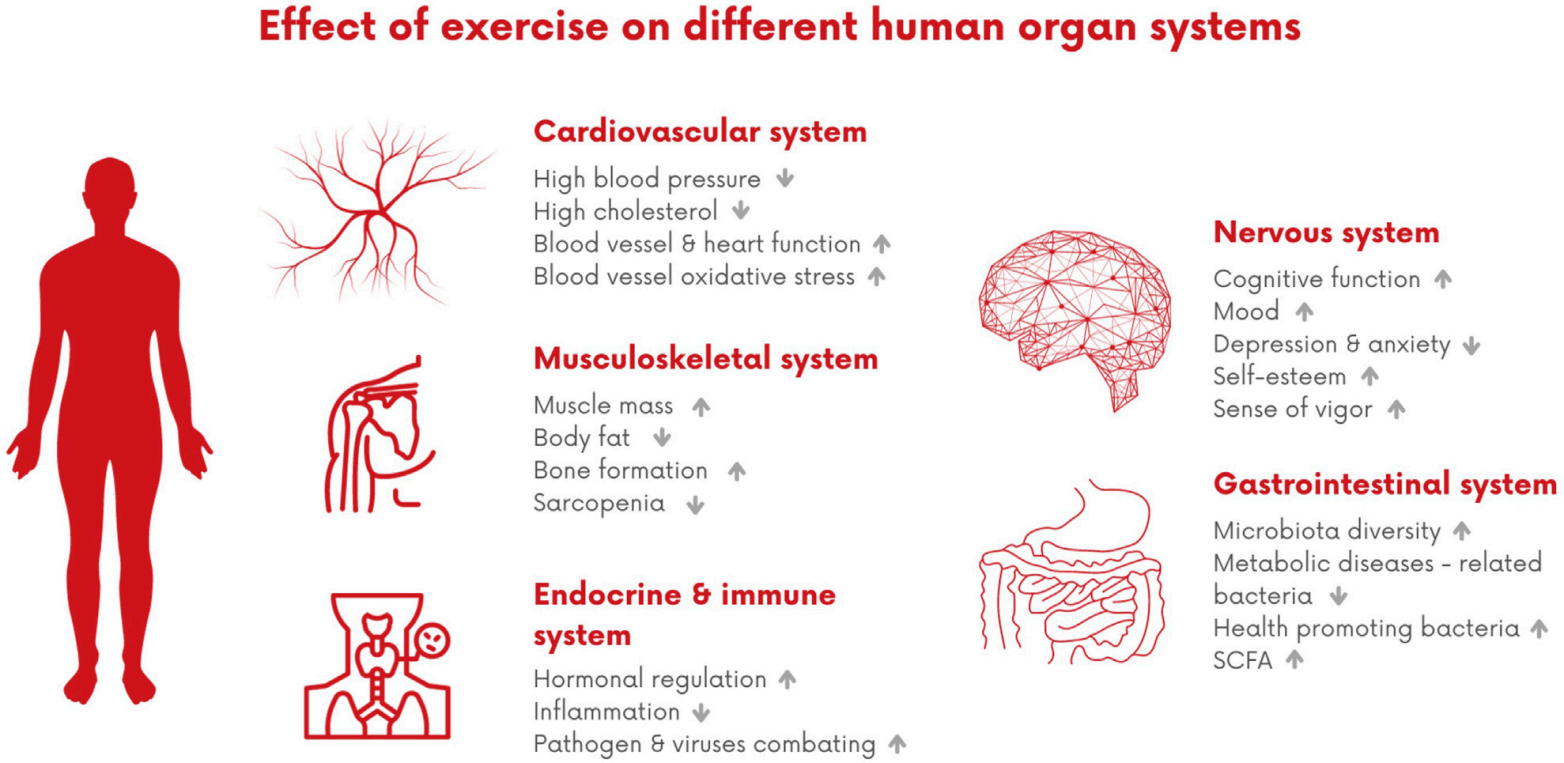
Effect of exercise on different human organ systems. This impact encompasses various benefits, including improvements in the cardiovascular system, such as enhanced blood vessels and heart function, and reduced levels of cholesterol. Additionally, it positively influences musculoskeletal health by increasing muscle mass and promoting bone formation. Furthermore, it contributes to improved hormonal regulation, decreased inflammation, and has positive effects on cognitive function and mood, while also aiding in reducing anxiety and depression. In terms of the gastrointestinal trait, it fosters microbiota diversity by promoting the growth of healthy bacteria.

In the following sections, we will emphasize the most important impact of exercise on some of the human organ systems related to aging and longevity and, ultimately, discuss the effects of exercise promoting longevity on the human body as a whole (Figure 2).

#### 3.1.1 Cardiovascular system

Exercise and physical activity have a profound influence on the cardiovascular (CV) system, and this plays a significant role in promoting longevity. Engaging in regular exercise and physical activity is recognized for its ability to reduce the risk of CV disease factors, including high blood pressure, elevated cholesterol levels, type 2 diabetes, and obesity, implying that such activities may contribute to preventing the accumulation of plaque in the blood vessels leading to atherosclerosis (Tian and Meng, 2019). Additionally, exercise and physical activity have direct positive effects on the structure and function of blood vessels and the heart, including cardiac preconditioning and improved autonomic balance (Fiuza-Luces et al., 2018). VO2 max, a measure of maximal oxygen consumption during intense exercise, is widely regarded as a strong predictor of longevity. Higher levels of VO2 max are associated with improved CV health, increased aerobic fitness, and a reduced risk of chronic diseases, contributing to a longer and healthier life (Letnes et al., 2023). One of the mechanisms by which exercise contributes to CV benefits is by improving the function of endothelial progenitor cells, which help repair damage to blood vessel linings (D’Onofrio et al., 2023). As people age, these cells tend to work less effectively, but exercise can enhance their function. Furthermore, exercise reduces oxidative stress in blood vessels associated with aging, enhances endothelial function, and boosts markers of blood vessel repair and the formation of new blood vessels (D’Onofrio et al., 2023). Epidemiological studies show that being physically active is associated with a reduced risk of death, whether someone has or does not have CV disease, with more significance for individuals with CV disease (Jeong et al., 2019). A systematic review and meta-analysis that included data from 21 studies and 159.352 CV disease patients showed that high cardiorespiratory fitness was associated with a 73% reduction in CV mortality risk and a 58% reduction in all-cause mortality risk (Ezzatvar et al., 2021). Furthermore, another meta-analysis that examined the relationship between step count and all-cause mortality or CV events found that an increase in 1000-step/day count was associated with a 23% reduction in all-cause mortality risk (Banach et al., 2023).

#### 3.1.2 Musculoskeletal system

The musculoskeletal system is composed of muscles, bones, joints, ligaments, tendons, fascia, and neuromuscular interface, which together support a person’s stability, mobility, and metabolism. Muscles are metabolically active organs that contribute to the proper functioning of insulin, glucose regulation, fatty acid oxidation, and other metabolic functions (Baskin et al., 2015). During aging, the musculoskeletal system structure and functions decline, which results in sarcopenia (loss of muscle mass, 3%–8% per decade after age 30, with an increased rate of decline after age 60), loss of muscle power and strength as well as a decrease in muscle flexibility and balance (D’Onofrio et al., 2023). Several age-related mechanisms are thought to be associated with sarcopenia, such as the decline in mitochondrial density and instability of mitochondrial DNA, dysregulation of the tissue renin-angiotensin system, as well as failure of adaptive responses to contractile activity is suspected to play a role in muscle mass loss, such as the ability to clear reactive oxygen species (Larsson et al., 2019; Saravi et al., 2021). Additionally, age-related changes include an increase in fat mass, a decline in basal metabolic rate (2%–3% per decade), and a decrease in bone mineral density (1%–3%/year) (Keys et al., 1973; Westcott, 2012). The aging process of the musculoskeletal system can be even more accelerated by the presence of other conditions and/or diseases, such as insulin resistance, diabetes, obesity, and chronic inflammation (Carapeto and Aguayo-Mazzucato, 2021).

There is wide evidence about the contribution of exercise, mainly strength and resistance training, in slowing the aging process of the musculoskeletal system. Resistance training promotes muscle growth and improves muscle quality by stimulating the motor unit, leading to the enlargement of muscle fibers, enhanced neuromuscular coordination, and increased strength. This is largely regulated by insulin, available amino acids, and the mTOR pathway (Atherton and Smith, 2012). Notably, there are only minimal reductions in muscle mass with age in the athletes (Wroblewski et al., 2011). Furthermore, chronically, the creation of higher muscle mass “speeds” metabolism by raising basal metabolic rate allowing individuals to burn more calories, which is important for managing weight, obesity and diabetes (D’Onofrio et al., 2023). One mechanism of exercise reducing sarcopenia is by decreasing inflammation and increasing anabolism and protein synthesis. Furthermore, activation of coactivators that are key regulators of mitochondrial biogenesis (e.g., PGC-1α), improves mitochondrial remodeling, muscle endurance, and enhanced balance and motor coordination in animal models (D’Onofrio et al., 2023).

Furthermore, resistance training is a long-term strategy for treating osteoporosis and age-related loss in bone mineral density. There is evidence to support that exercise leads to significant changes in bone mineral density, decreases in total body fat mass, and increases in total body lean mass (Santos et al., 2017). Resistance training also prevents fall risk among older adults (Carter et al., 2002). One explanation could be that weight-bearing activities can induce bone formation by delaying telomere shortening and modifying DNA methylation (Carter et al., 2002).

#### 3.1.3 Immune and endocrine system

Immunosenescence is the gradual deterioration of the immune system with age, characterized by significant shifts in the balance of leukocyte subsets. Regular exercise may help slow down the immunosenescence process by promoting the health and function of immune cells (Weyh et al., 2020). For example, long-term exercise from adulthood to old age induces anti-inflammatory effects, which is indicated by decreased level of inflammatory biomarkers, including white blood cell and neutrophil counts, IL-6, IL-10, IL-1 receptor antagonist, and soluble tumor necrosis factor receptor-1 (Moore et al., 2012).

Furthermore, aging weakens the immune system’s ability to fight infections, including viruses and bacteria, and exercises emerge as a safe and cost-effective strategy for combating infectious diseases. For example, it was shown that a regular program of aerobic exercise (intensity of 55%, 80% VO2max) helped to reduce the risk of coronavirus disease (COVID-19) by increasing immunological biomarkers, such as circulating lymphocytes, leukocytes, monocytes, and neutrophils (Abdelbasset, 2020; Alawna et al., 2020; Mohammed et al., 2021).

Moreover, regular exercise profoundly affects the endocrine system, playing a role in longevity by regulating hormones and diminishing chronic inflammation. Notably, exercise positively influences insulin interactions, enhancing its efficiency in transporting glucose into muscle cells. This process helps regulate blood sugar levels, reducing the reliance on excessive insulin production and decreasing insulin resistance (Yaribeygi et al., 2019).

#### 3.1.4 Gastrointestinal system: gut microbiota

In recent years, gut microbiota and intestinal health have been described as important players shaping overall human health, and gut dysbiosis has been regarded as a new hallmark of aging (López-Otín et al., 2023). The beneficial effect of exercise on gut microbiota is contributed by several mechanisms, including an increase in bacterial diversity (usually associated with favorable health outcomes), an increase in fecal short chain fatty acids (SCFA), an increase in the proportion of health-promoting bacterial species (e.g., *Faecalibacterium prausnitzii*, and *Akkermansia muciniphila*), and a decrease of obesity and metabolic diseases-related bacteria (e.g., *Eubacterium rectale* and *Clostridium coccoide*a) (Estaki et al., 2016; Bressa et al., 2017; Allen et al., 2018; Yuan et al., 2022). A systematic review showed that aerobic training (≥2 weeks) of at least 60 min in non-athlete subjects increased diversity indexes of gut microbiota (Dziewiecka et al., 2022). Also, it was shown that athletes usually have more diverse microbiota, a higher abundance of SCFA, and lactic acid-producing bacteria. Additionally, the study showed that for inducing the changes in gut microbiota not only dose and mode of exercises are important, but also the patterns such as breaks in sedentary time and avoidance of long periods of inactivity in daily life (Dziewiecka et al., 2022).

#### 3.1.5 Nervous system: brain, cognitive health, and wellbeing

Consistent exercise could enhance cognitive functions and protect against the onset of cognitive decline or dementia. In a study of 900 older individuals engaging in resistance training exercises, an increase in muscle strength correlated with a remarkable 43% decrease in the risk of developing Alzheimer’s disease and a 33% reduction in the risk of mild cognitive impairments (Boyle et al., 2009), exercise is associated with psychological benefits, including more positive mood, reduction in depression and anxiety, higher self-esteem, and a sense of vigor (Gordon et al., 2017; Amiri, 2023). In a comprehensive meta-analysis encompassing 33 randomized clinical trials and 1877 participants, resistance training demonstrated a significant reduction in symptoms of depression across various baseline health statuses, indicating a moderate effect size (Gordon et al., 2018).

#### 3.1.6 Systemic effect

The human body functions as a unified system. Therefore, a single exercise intervention, such as resistance training, can yield multiple benefits for various organs and systems within the body. For example, muscle, which belongs to the musculoskeletal system, is also an endocrine organ, producing and releasing signaling molecules (myokines) into the bloodstream in response to muscle contractions and exercise. Myokines may directly enhance CV health by improving endothelial cell function, protecting against endothelial injury and atherosclerosis, and positively impacting myocardial contractility (Pedersen and Febbraio, 2012). Furthermore, the effects of exercise on muscle health may play an important role in bone health and other organs and tissues. For example, the protein irisin secreted by skeletal muscle can regulate bone remodeling but also affects adipose tissue, cholesterol metabolism, inflammation, glucose tolerance, and cognitive function (D’Onofrio et al., 2023). Also, the beneficial effect of exercise on muscle mass is known to help in the management of insulin resistance, which is a prediabetes state, thus preventing type 2 diabetes by having the so-called “browning” effect of white adipose tissue and effect on cellular energetics through mitochondrial biogenesis (Weyh et al., 2020).

Further, regular exercise positively impacts the gut and produces systemic benefits. For example, exercise protects intestinal permeability, reducing the absorption of molecules like lipopolysaccharide (LPS) that contribute to systemic inflammation—another factor associated with CV disease, obesity, and yype 2 diabetes. This protective effect helps prevent the initiation of pro-inflammatory responses and the formation of macrophage cells, which are early indicators of atherosclerotic lesions (Yuan et al., 2022; D’Onofrio et al., 2023).

#### 3.1.7 Guidelines, protocols, and recommendations

The evidence in previous sections clearly suggests that exercise and physical activity benefit human health and lifespan. Regarding guidelines and protocols, the scientific community generally accepts the World Health Organization (WHO) protocols.

According to the WHO, for adults (18–64 years old), physical activity is regarded as critical for reducing all-cause mortality; managing weight; reducing adverse events; improving cognitive outcomes and mental health; as well as reducing the incidence of type 2 diabetes, CV diseases, and cancer (Bull et al., 2020). The recommended weekly activity is 150–300 min of moderate-intensity aerobic exercise or 75–150 min of vigorous-intensity activity, with higher duration and intensity enhancing CV and metabolic effects. A recent study confirmed that adhering to these guidelines reduced the risk of early death by up to 25% and that exceeding recommendations by 2–4 times further lowered the risk by an additional 4%–13% (Lee et al., 2022). Aerobic physical activity beyond 300 min/week of moderate intensity or 150 min/week of vigorous intensity is conditionally beneficial, as the optimal threshold remains unclear. As early as 1986, studies reported a reverse J-shaped association between exercise (e.g., walking, stair climbing, sports play) and all-cause mortality. This curve suggests a dose-dependent relationship between exercise and longevity until an optimal threshold is surpassed, beyond which benefits may decline (O’Keefe et al., 2020). Some research also suggests that prolonged exercises exceeding 10 h/week might have undesirable effects (O’Keefe et al., 2020).

In children (5–17 years old), the WHO underscores the importance of physical activity for bone health and the development of prosocial behavior, both important factors to longevity. Physical activity is recommended for older adults to prevent falls, fall-related injuries, and declines in bone health and functional ability. In addition to aerobic activities, adults 65 years of age and older are advised to engage in multicomponent physical activities, encompassing functional balance and strength training at a moderate or higher intensity for at least 3 days a week (Bull et al., 2020).

These recommendations are primarily derived from observational studies. To establish more precise guidelines, the scientific community should: (i) Establish protocols for conducting high-quality randomized controlled trials (RCT); (ii) Define universal cut-points for classifying different intensity intervals. Intensity is measured using metrics like %VO2max (% of maximal oxygen consumption) or %VO2R (% oxygen uptake reserve), with various cut-points in studies. Other physiological variables (maximum heart rate, heart rate reserve, ventilatory or lactate thresholds) may also be used to identify intensity zones; (iii) Develop more accurate technologies (e.g., smartwatch tools); and foster a consensus among researchers on classifying individuals into different intensity levels.

Despite these challenges, the overall evidence implicate the following: Physical activity and exercise, as a dynamic and energy-intensive activity, impart a plethora of benefits to the CV, musculoskeletal, immune, endocrine systems, and gastrointestinal systems, all contributing to longevity and overall wellbeing. It is recommended that individuals of all ages should adhere to the minimum guidelines for physical activity to promote longevity. Starting from an early age, consistent exercise should be integrated into daily routines. Middle-aged and older individuals, if not already engaged, should begin incorporating exercise.

### 3.2 Dietary interventions

The relationship between dietary patterns and longevity is nowadays a subject of profound scientific interest, including various dietary strategies that influence the aging process and overall health. In the following sections, we will explore dietary interventions known for their potential impact on longevity.

#### 3.2.1 Caloric restriction

Caloric restriction (CR) is the most robust and the best studied dietary intervention for its effect on promoting longevity. Already in 1935, it was noted that CR could prolong the lifespan in rats, and since then, scientific evidence showing the positive benefits of CR both in animal models and in humans has been accumulating (McDonald and Ramsey, 2010). CR includes reducing calorie intake over a given period while maintaining essential nutrients. It involves consuming fewer calories than what is considered the standard daily energy requirement for an individual’s sex, age, and activity level (Dorling et al., 2020). Typically, in humans, the most studied is 10%–25% CR per day. CALERIE (Comprehensive Assessment of Long-term Effects of Reducing Energy) I and II were the first controlled clinical trials to assess the efficacy, safety, and feasibility of a 6–12 months CR diet. CALERIE I trial showed that CR improves biomarkers associated with aging and age-related diseases, including a reduction in markers of energy metabolism metabolic adaptation and improvements in many CV risk factors. This trial also showed that a moderate 12%–18% CR is safe and feasible, not compromising quality of life (Rickman et al., 2011).

CALERIE II study aimed to explore the effects of prolonged CR durations related to an organism’s lifespan and establish if CR-induced alterations have an effect beyond the initial period of weight loss. The study showed that after the period of 2 years, an average of 11.7% CR could improve longevity and decelerate primary aging by decreasing the mass-specific metabolic rate and total energy expenditure. By lowering energy expenditure there is a decrease in the production of reactive oxygen species and DNA damages, proteins, and lipids crucial for normal cellular function. Furthermore, healthy, and young participants significantly improved body composition, inflammatory and cardiometabolic risk biomarkers, and aerobic fitness (Dorling et al., 2021). This suggests that CR slows secondary aging by improving numerous markers of disease risks.

The benefits of CR are also confirmed in the Biosphere 2 experiment that involved individuals who lived in a closed ecological system for 2 years. Although not explicitly intended for the investigation of CR, the circumstances within a closed ecological setting naturally induced CR due to the limited food resources. The study provided insights into the positive effects of CR on human physiology, including changes in metabolism and body weight (Walford et al., 1992).

Various studies in animal models and humans showed that CR activates multiple molecular pathways that promote genome stability, proteostasis, stress resistance, and stem cell function mainly by inhibiting key nutrient-sensing and inflammatory pathways (Most et al., 2017; Kökten et al., 2021). CR involves multiple neural, tissue-specific, cell-autonomous, and systemic mechanisms that are the sum of various metabolic, transcriptional, proteomic, and even microbiota alterations (Dorling et al., 2020). Furthermore, as changes occur at varying levels and in different pathways that result in enhancement of neurogenesis, synaptic plasticity, brain structure, and function, CR has also been shown to be beneficial for cognitive protection and improvement of slowing down the progression of cognitive age-related diseases such as Alzheimer’s disease (Van Cauwenberghe et al., 2016; Alharbi et al., 2023; Rahmani et al., 2023).

While considerable beneficial evidence exists in both human and animal studies supporting CR, certain challenges may arise during prolonged CR. Such challenges include decreased adherence, feelings of hunger, weight regain, and potential loss of lean mass and bone density (Hofer et al., 2022). However, data on these outcomes are inconsistent, necessitating further research. These effects can be mitigated with careful consideration of intervention duration, nutrient composition, and complementary exercises. Individual responses to CR, optimal levels, and durations require a personalized approach. In the current clinical practice, CR is recommended for weight management. Nevertheless, based on scientific evidence from pre-clinical and human studies, future clinical applications may extend to recommending CR for specific pathological conditions such as CV diseases, metabolic disorders, and cancers.

#### 3.2.2 Intermediate fasting

Unlike CR, where there is a constant reduction of meal size and/or calorie intake during the meals, intermediate fasting (IF) is characterized by temporal dietary restriction by skipping one or more consecutive meals (Varady et al., 2022).

There are various types of IF differing in rhythmic or arhythmic regimens, including Alternate Day Fasting (ADF), individual fasts every other day, without any calorie intake or if mildly performed, 25% of baseline calorie intake is permitted; Periodic Fasting (PF) where individual can follow different schemes such as so-called 5:2, where calories are restricted in 2 days a week to a minimal level; Time-Restricted Eating (TRE) where individual skip a specific meal (breakfast or dinner), or by consuming the meals into a narrow time window. The detailed health outcomes of different IF regimes through different studies are very well reviewed and described by Dorling and coauthors (2020).

In general, alike in CR, IF decreases markers of systemic inflammation (that, among other reasons, may be due to the chronic excess calorie consumption) and oxidative stress that are two important drivers of aging and metabolic diseases such as T2D, insulin resistance (Meydani et al., 2016; Stekovic et al., 2019). Both IF and CR improve the blood lipid profile and other relevant factors for CV disease (Most et al., 2017; Trepanowski et al., 2018; De Cabo and Mattson, 2019), animal models of neurodegenerative diseases, such as Alzheimer’s IF and CR, were able to delay the progression of phenotypes of the disease (Mattson et al., 2017). Marinac et al. (2016) showed that more than 13 h of fasting during nighttime is associated with reduced breast cancer risk. Also, a recent systematic review concluded that different IF regimes could positively impact the diversity and abundance of gut microbiota (Pérez-Gerdel et al., 2023).

Regarding the mechanisms, prolonged periods of fasting state are characterized by a metabolic switch from glucose dependency to elevation of lipid metabolism, including free fatty acid usage and production of ketone bodies in the liver (Hofer et al., 2022). This, in turn, stimulates adaptive processes, improving body composition and physiological function. The process of metabolic switching usually starts after 12–36 h of food abstinence, but this depends on many factors, such as baseline hepatic glycogen storage and activity levels. Cells sense nutrient levels and adjust their behavior through various interconnected pathways, including activation of AMPK and shutdown of mTOR pathway and engagement of various transcription factors (e.g., FOXOs, PGC-1α), resulting in activation of autophagy, mitophagy DNA repair, and oxidative stress defense (Hofer et al., 2022).

Other types of fasting or CR-mimicking interventions include various dietary interventions aiming to elicit some CR-like molecular and physiological effects, such as fasting-mimicking diets, low-carbohydrate/fat diets, or ketogenic diets (Brandhorst et al., 2015; Boison, 2017; Kim, 2021).

#### 3.2.3 Plant-based diets

Within plant-based diets various dietary patterns are focusing on plant-based food sources with little or no consumption of animal products, thus up to date there is no one universal definition for plant-based foods. The most practiced plant-based diets include vegetarian diet (diet that excludes meat and poultry), and vegan diet (diet that excludes any type of food derived from animals, e.g., meat, dairy products, honey).

In recent years, there has been an increased interest in potential longevity-promoting pathways associated with plant-based diets. These diets, characterized by restricted protein intake, amino acids such as branched-chain amino acids (BCAA) and methionine, share the same mechanisms with the mechanisms linked to health improvement and longevity observed in CR diets (Herpich et al., 2022). The Adventist Health Study 2 (AHS-2) with 96.000 adults found that ovo-lacto-vegetarian diets (includes eggs, dairy, and honey, but not fish) were linked to lower all-cause mortality, BMI, and reduced risks of diabetes, hypertension, and certain cancers (Orlich et al., 2013). Vegan diets showed even lower risks for CV mortality, obesity, hypertension, and type 2 diabetes. Herpich and colleagues (2022) conducted a comprehensive meta-study on plant-based diets, revealing diverse outcomes regarding mortality reduction and health improvements. These findings are dependent on the inherent variations among plant-based diet types and are not universally applicable across different plant-based diet categories. Future large-scale human studies are warranted to understand better the specific mechanisms influencing longevity. Nevertheless, well-planned plant-based diets provide metabolic, CV, and intestinal benefits, ultimately contributing to an extended health span and potentially an increased life span.

#### 3.2.4 Mediterranean diet

The Mediterranean diet (MedDiet) reflects dietary patterns traditionally observed in civilizations cultivating olives in the Mediterranean region, particularly in southern Italy, Greece, and Crete. Key features of the MedDiet include elevated consumption of virgin and extra virgin olive oil as the primary fat source, along with a substantial intake of vegetables, legumes, fruits, minimally refined cereals, nuts, seeds, and fresh, locally sourced, seasonal, and minimally processed foods. Also, the diet involves limited or moderate intake of dairy products, fish, and poultry, while red meat and sweets are consumed sparingly. Additionally, moderate wine consumption is included during meals Andreo-López et al., 2023).

MedDiet has been associated with low rates of age-related diseases, mainly CV, metabolic, neurodegenerative, and oncological conditions, contributing to an extended life expectancy (Martinez-Gonzalez and Martin-Calvo, 2016). The constituents of the MedDiet, such as essential omega-3 and 6 fatty acids, polyphenols, fibers, and vitamins, are reported to positively impact the molecular pathways associated with all recognized hallmarks of aging (Martinez-Gonzalez and Martin-Calvo, 2016; Campanella et al., 2021). Future studies should provide a better understanding of the mechanism of action of MedDiet on aging as a whole. However, the benefits of MedDiet constituents on human health present robust evidence; thus, this diet could be practiced delaying age-related diseases and maintain good health.

#### 3.2.5 “Longevity diet” by Valter Longo, Ph.D

This diet is based on the pillars from previously mentioned findings of the life span research in model organisms and clinical studies (Longo and Anderson, 2022). It is developed by biochemist Valter Longo, Ph. D, and below are the main characteristics of this diet:

• Periodic fasting or calorie restriction have a central role;
• Strong emphasis on plant-based foods, including fruits, vegetables, legumes, and whole grains;
• Moderate protein intake, with an emphasis on plant-based protein sources, that is contrary to high-protein diets that promote significant animal protein consumption (e.g., keto-diet);
• Consumption of healthy fats (unsaturated and polyunsaturated), such as those found in nuts, seeds, and olive oil, and avoiding saturated fats;
• Consumption of whole, unprocessed foods and exclusion of processed and refined foods, as well as red and processed meats;

Additionally, the author emphasizes the necessity of tailoring the diet to age, sex, and health status to prevent malnutrition. Ongoing research on the longevity diet persists, with the scientific community actively investigating its potential benefits for health and longevity.

#### 3.2.6 Blue zones diets

Blue zones are the geographical areas characterized by exceptionally long-lived populations. They include the island of Ikaria in Greece, the island of Okinawa in Japan, the mountain area of Sardinia in Italy, and the peninsula of Nicoya in Costa Rica. Their diets share some common patterns with all previously described diets, such as predominant consumption of plant-based foods, moderate protein intake, intake of healthy fats, and caloric moderation (Kreouzi et al., 2022). Since these populations have been long-lived, researchers started investigating the Blue Zones’ eating regimes.

Nevertheless, there is a common tendency to assume that the mere documentation of a diet within a long-lived population automatically establishes it as a causal factor for the population’s longevity. Such a claim requires substantiation and should be supported by evidence. It is important to note that diet is just one component of the broader lifestyle factors associated with longevity in Blue Zones. Other factors include physical activity, strong social connections, a sense of purpose, and a low-stress lifestyle, which also confirms that multiple aspects of live should be satisfied for longevity.

Additionally, recently significant question about the validity of Blue Zones raised, especially one by Dr Saule Newman, an Oxford researcher (Newman, 2019). He argues that longevity observed in these regions may be due to inaccurate record-keeping and age exaggeration rather than from unique lifestyle factors, as well as historical gaps in vital records leading to overrepresentation of centenarians. His analysis calls for a more cautious interpretation of Blue Zones, where more scientific studies are needed to verify these claims and clarify whether the Blue Zone lifestyle truly accounts for extended lifespans.

### 3.3 Oral health

The concept of ‘oral frailty,’ recently introduced in Japan, is reshaping our understanding of oral health’s impact on longevity (Watanabe et al., 2020). Oral frailty, characterized by diminished oral function in tandem with mental and physical decline, has been linked to an increased risk of physical frailty, sarcopenia, severe health conditions requiring nursing care, and mortality (Tanaka et al., 2018). Notably, the traditional focus on the number of teeth as an oral health indicator is evolving to encompass overall oral function. This shift is particularly relevant in the context of longer life expectancies and better dental care, leading to longer retention of natural teeth (Fukai et al., 2017).

Research has elucidated the relationships between the number of teeth, dentures, occlusion, and health outcomes (Shimazaki et al., 2001; Holm-Pedersen et al., 2008; Polzer et al., 2012; Kaufman et al., 2014; Matsuyama et al., 2017). For example, individuals retaining more than 20 teeth have demonstrated significantly lower mortality risks and reduced nursing care needs compared to those with fewer or no teeth (Watanabe et al., 2020). Furthermore, maintaining or enhancing oral function, coupled with a nutritious diet, is increasingly recognized as essential for overall health. Diminished oral function is a major risk factor for malnutrition and sarcopenia (Dibello et al., 2021).

The concept of oral frailty is influencing dental and oral health policies, especially in aging societies like Japan (Watanabe et al., 2020). This concept integrates various oral conditions, including tooth count, oral hygiene, and oral functions, and correlates with declines in physical and mental health (Liu et al., 2011; Liu et al., 2022). Additionally, research has shown a direct impact of oral functions, such as mastication and swallowing, on life expectancy. Higher subjective masticatory function has been associated with longer life spans (Watanabe et al., 2020).

Dental caries and periodontitis, by triggering and aggravating inflammatory states, are not only linked to systemic diseases but also contribute significantly to the global health burden (Saravi et al., 2020; Botelho et al., 2022; Vollmer et al., 2022). Together, they represent around 23.1 million disability-adjusted life-years (DALYs) globally, which translates to a loss of approximately 284.6 years per 100,000 population, with 28.02% of this increase in DALYs between 1990 and 2010 attributed to the aging population (Vollset et al., 2020; Tu et al., 2023). This underscores the urgent need for radical reforms in dental care, especially considering the growing number of people with oral disorders due to population aging.

The World Dental Federation’s 2016 redefinition of oral health broadens the scope beyond disease to include essential functions like speaking, smiling, and eating, highlighting the substantial impact of oral health on overall quality of life. Suboptimal oral health, manifested through issues like broken or missing teeth, can lead to oral pain, infections, reduced nutritional intake, and difficulties in communication and mastication, significantly affecting daily living (Glick et al., 2016; Wu et al., 2018).

Given these challenges, it is imperative to reorient healthy aging policies to prioritize oral health. Adopting the “*Health in All Policies*” approach can facilitate this by embedding oral health into broader national governance frameworks, encouraging collaborative policymaking across different sectors. This approach has proven effective in various national contexts, demonstrating that clear understanding between health and other policy areas leads to more proactive health prevention measures (Patel et al., 2021). The WHO Integrated Care for Older People (ICOPE) guidelines offer a proactive vision for monitoring health changes in older adults, focusing on intrinsic capacity. By incorporating these guidelines, national policies can better support healthy aging and integrate health and social care systems. Implementing oral health services within this integrated health system can contribute significantly to achieving universal health coverage and addressing the needs of those at risk of substantial loss of intrinsic capacity or functional ability. The ICOPE framework’s emphasis on intrinsic capacity reinforces the importance of routine oral health assessments, recognizing oral health as a vital component of older adults’ overall health (Patel et al., 2021).

### 3.4 Multifaceted aspects shaping longevity

**FIGURE 3:**
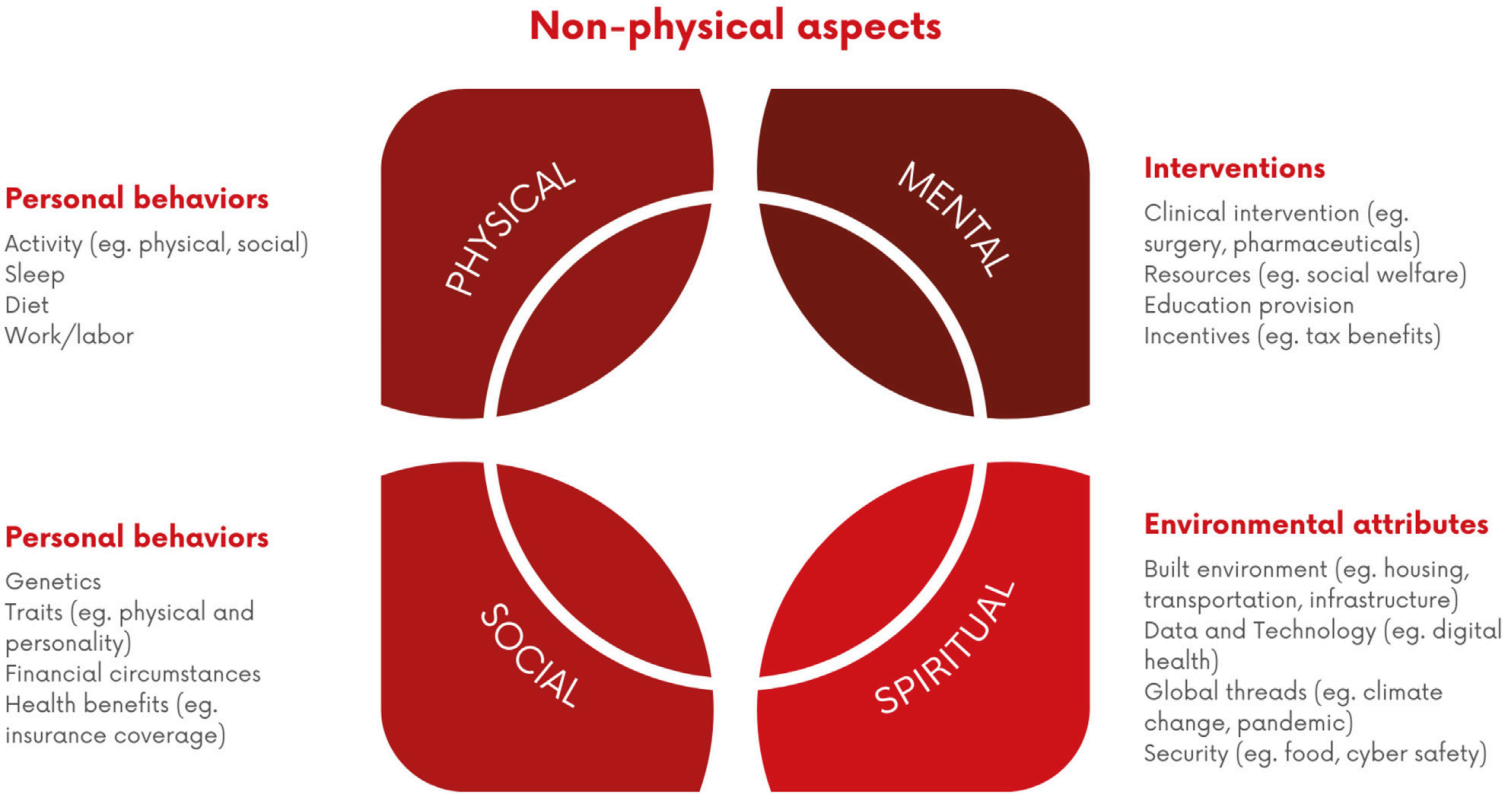
Interconnected Factors Influencing Longevity. A visual representation of the interrelated non-physical factors contributing to longevity, categorized into personal behaviors (e.g., activity, sleep, diet), personal attributes (e.g., genetics, health benefits), interventions (e.g., clinical, educational), and environmental attributes (e.g., housing, security).

Longevity is influenced by a combination of interconnected factors that encompass various aspects of an individual’s life. This includes genetics, lifestyle choices and various environmental factors which are identified as physical, social, mental, and spiritual aspects (Wang et al., 2023) (Figure 3). It is essential to recognise the interplay between genetics and lifestyle choices (e.g., avoidance of ultra-processed foods) and focus on holistic health approaches. Adopting healthy lifestyles such as ones already described in previous paragraphs can have a substantial impact on overall health and longevity, even for individuals that have some adverse genetic predispositions (Wang et al., 2023). Furthermore, the environment in which people live such as access to clean air and water, pollutants exposure, availability of green spaces, the quality of housing, as well as the access to healthcare services can significantly influence health and lifespan (Queen et al., 2020). Therefore, public, and private health interventions, urban planning prioritizing human wellbeing, opening of new longevity clinics, as well as policies aimed at reducing environmental pollution all play an important role in creating environments for longer and healthier lives.

## 4 Dietary supplements

### 4.1 NAD + precursors

Nicotinamide adenine dinucleotide (NAD+) is a coenzyme that plays a crucial role in various cellular processes, including energy metabolism, immune function regulation, and DNA repair. NAD + level in cells is associated with aging-related processes as its level declines during aging (Sharma et al., 2023). Over the last decade, the interest in NAD + can be attributed to its ability to activate the family of NAD + -dependent proteins called sirtuins. Sirtuins have been shown to play a major regulatory role in almost all cellular functions, and they impact inflammation, energy metabolism, cell growth, circadian rhythm, neuronal function, and stress resistance (Rajman et al., 2018). Two main NAD + precursors, nicotinamide riboside (NR) and nicotinamide mononucleotide (NMN) can be converted into NAD+ in the body. Therefore, supplementation with NAD + precursors could be a good strategy for disease prevention and increased longevity.

NR, known as niagen, is a form of vitamin B3. NR supplementation using a single oral 1,000 mg dose showed the ability to raise 2.7-fold NAD+ in human blood (Trammell et al., 2016). Another study showed that NR oral administration increased NAD+ in a dose-dependent manner in the range of 250–1,000 mg/day (Airhart et al., 2017). In humans, NR has positive effects on vascular endothelial function, increased number of mitochondria, and improved gut microbiota composition in healthy adults (Rajman et al., 2018; Lapatto et al., 2023). Also, there is a vast amount of evidence in animal models showing NR has a positive effect on energy metabolism and brown fat, slowing certain types of acute and chronic kidney diseases and preventing cognitive decline (Rajman et al., 2018). Future studies are needed to confirm these effects in humans.

NMN is involved in the same NAD + biosynthetic pathway as NR, namely, the NR kinase pathway. Up to now, in humans, it was shown that NMN was able to increase muscle insulin sensitivity in prediabetic women, improve muscle aerobic capacity in amateur runners, and improve fatigue in older adults (Liao et al., 2021; Yoshino et al., 2021; Kim et al., 2022). The results from preclinical studies such as the ability of NMN to improve diabetes induced memory deficits, prevent heart failure, improve cognition, and prevent infertility (Rajman et al., 2018) should be further confirmed in humans.

Both NR and NMN are safe and well tolerated by humans in dosages 100–2000 mg, and 250–1,000 mg/day, respectively (Airhart et al., 2017; Pencina et al., 2023). Additionally, the timing of NAD + precursor consumption has demonstrated significance, as studies indicate that the metabolism level of NAD+ and SIRT1 activity peaks at noon every day (Sadria and Layton, 2021). Therefore, supplementing with NAD+ during this peak period could optimize NAD + utilization.

### 4.2 Spermidine

Spermidine is a polyamine that can be naturally produced by human cells or by the gut microbiota. spermidine from food sources including nuts, wheat germ, and soybeans has a good bioavailability (Madeo et al., 2018). The same as NAD+, the level of spermidine declines with aging. Elevated serum spermidine levels have been linked to longevity in healthy centenarians (Pucciarelli et al., 2012). Studies in humans have shown that spermidine can improve cognitive decline in senior citizens at risk of dementia (Wirth et al., 2018) and is associated with a reduced risk of CV diseases (Eisenberg et al., 2016). Furthermore, supplementation with spermidine has demonstrated the potential to extend lifespan across various species, foster cardiovascular health, provide neuroprotection, and enhance skeletal muscle regeneration (Madeo et al., 2018).

The average daily spermidine intake is 7–25 mg or more, with the highest reported levels found in the MedDiet (Madeo et al., 2018). Most supplements available on the market contain spermidine dosages within this range.

### 4.3 Alpha-ketoglutarate

Alpha-ketoglutarate (AKG) is a molecule naturally present in our cells. It is an intermediate compound in the tricarboxylic acid (TCA) cycle, playing a crucial role in cellular energy metabolism. AKG is closely linked to amino acid metabolism; it serves as a precursor to the amino acids glutamate and glutamine, which have various roles in cellular function, including neurotransmission and nitrogen transport (Gyanwali et al., 2022). Like NAD+ and spermidine, the levels of AKG decline as we age. Research on AKG and its potential impact on human longevity is an area of growing interest since it showed the ability to increase lifespan in various model organisms. AKG showed the potential to suppress inflammation, reduce phenotypes of senescence cells, improve bone mass, maintain gut integrity, and induce fat browning (Chitalia, 2014; Chen et al., 2017; Asadi Shahmirzadi et al., 2020). In humans, AKG supplementation is mainly used for improving sports performance, and some of the studies showed improvements in specific exercises such as bench press and Wingate anaerobic test performance (Campbell et al., 2006). Furthermore, in a retrospective human study, a commercially available supplement containing AKG reversed biological age up to 7 years on average (Demidenko et al., 2021). Skin health is also important for longevity, and AKG stimulates collagen synthesis and proteins for optimal detoxification and significantly reduced wrinkles (Gyanwali et al., 2022; Yang et al., 2022).

Most available supplements on the market contain from 300 to 1,500 mg per dosage of arginine or calcium-alpha ketoglutarate per dosage. In the future, long-term clinical studies are required to establish AKG-specific mechanisms and effects on human longevity.

### 4.4 Resveratrol

Resveratrol is a natural polyphenol found in certain plants, including the skin of red grapes, peanuts, and berries, as well as in red wine (Meng et al., 2020). As the amounts present in these sources may be relatively low to induce longevity-related health effects, supplementation is needed. The anti-aging potential of resveratrol is due to its antioxidant and anti-inflammatory properties (Meng et al., 2020). As for NAD + precursors, the proposed mechanism through which resveratrol may impact longevity is activating sirtuins (Meng et al., 2020). In humans, resveratrol improves glycemic control in type 2 diabetes people and may cause some fat loss (Timmers et al., 2011; Hoseini et al., 2019). The results from *in vitro* studies suggest that it is beneficial for atherosclerosis prevention and modest enhancements for vasorelaxation and blood flow (Mizutani et al., 2000; Rush et al., 2007). It was proposed that these cardioprotective effects are due to its inhibition of pro-hypertrophic signaling molecules, improvement of myocardial calcium handling, and reduction of oxidative stress and inflammation (Salehi et al., 2018).

In healthy individuals, dosages of up to 5 g per day were shown to be safe and well-tolerated (Salehi et al., 2018). Most available supplements on the market contain resveratrol dosages of 100–500 mg per day as trans-resveratrol. Resveratrol shows promise; however more rigorous long-term human studies are needed.

### 4.5 Senolytic supplements: fisetin & quercetin

#### 4.5.1 Fisetin

Fisetin is a flavonoid found in various fruits and vegetables, such as strawberries, apples, onions, and cucumbers. Fisetin is known for its antioxidant, anti-inflammatory, anticancer, and neuroprotective properties. The key mechanism through which fisetin promotes longevity is its senolytic activity (Khan et al., 2013). It means that fisetin can remove accumulated senescence cells from the body that damage neighboring cells and tissues, which induces inflammatory damage and leads to various age-related diseases. When tested together with other flavonoids in mice and in senescent human fibroblasts, fisetin showed to be the most potent senolytic (Yousefzadeh et al., 2018). In model organisms, fisetin showed the ability to prolong lifespan, particularly 13% in mice, 23% in *Drosophila*, and 55% in *Saccharomyces cerevisiae* (Khan et al., 2013; Yousefzadeh et al., 2018). Furthermore, it showed the potential to reduce neurodegenerative diseases and prevent certain types of cancer (Khan et al., 2013). Given Fisetin’s potential in combating age-related diseases, ongoing clinical trials are exploring its efficacy against inflammation, CV diseases, certain cancers, and in reducing mortality and long-term complications associated with COVID-19.

Most of the studies used 20–500 mg per day, with no significant adverse effects reported; also available supplements on the market contain fisetin dosages within this range.

#### 4.5.2 Quercetin

Quercetin is another flavonoid compound found in many fruits, vegetables, and grains. Quercetin is known for its cardioprotective, anti-inflammatory, antiviral properties, and anti-cancerogenic activities (Aghababaei and Hadidi, 2023) all important for longevity. Laboratory aging models demonstrate that quercetin increases lifespan by up to 60% (Belinha et al., 2007). In humans, quercetin showed its potential to protect against CV diseases (Egert et al., 2009; Pfeuffer et al., 2013). In combination with dasatinib, it can selectively decrease the number of naturally occurring senescent cells, as well as reduce adipose tissue inflammation, and improve systemic metabolic function in old age (Zhu et al., 2016; Islam et al., 2023). Furthermore, intermittent oral administration of these two compounds reduced mice mortality hazard by up to 65% (Xu et al., 2018).

Dosages of quercetin up to 1 g are well tolerated. It is suggested to administer quercetin supplements with other bioflavonoids such as resveratrol, genistein, or green tea catechins to increase bioavailability. The best bioavailability form showed to be dihydrate, followed by glycosides, aglycone, and rutinoside (Jaffe and Mani, 2018).

### 4.6 Probiotics

Already in the early 1900s, Elie Metchnikoff proposed the idea that aging might be slowed down by enhancing the intestinal microbiome through the consumption of yogurt-enriched with beneficial bacteria. The communication between the gut microbiota and various host organs, termed the “*Gut-X*″ signaling axis (e.g., gut-liver, gut-bone, and gut-brain axes), significantly influence human health (Sun et al., 2023). Consequently, strategies targeting intestinal microbial homeostasis are emerging as crucial topics in the study of aging and age-related pathologies. Microbiome dysbiosis sets off a cascade of inflammatory and pathological events, all strongly linked to age-related diseases. Probiotics, as beneficial bacteria, are known to regulate the gut microbiota, thus, recently, they have been seen as promising anti-aging approaches (Duan et al., 2022).

The abundance of several beneficial bacteria, such as *Bifidobacterium* spp., *Lactobacillus* spp., and *Akkermansia* spp., decreases with age. Supplementing these bacteria through probiotics has the potential to slow down the aging process and enhance various age-related symptoms. Also, next-generation probiotics are showing promising results in animal models. Specifically, *Propionibacterium freudenreichii* was able to increase the lifespan of *Caenorhabditis elegans* (Kwon et al., 2016), and supplementation of *A. muciniphila*, the species that decreased in old mice, improved the intestinal senescence-related phenotype, and extended the healthspan (Duan et al., 2022). Recently the term “*gerobiotics*'' is defined for the probiotic strains and their derived postbiotics and para-probiotics able to beneficially attenuate the fundamental mechanisms of aging, reduce physiological aging processes, and thereby expand the host health span (Tsai et al., 2021). Probiotics would be registered under this definition if the ability to extend the lifespan is proven in preclinical and clinical studies (Liu et al., 2011). Aa a promising gerobiotic candidate is proposed the *Lacticaseibacillus paracasei* PS23, which has shown the ability to reduce the age-related loss of muscle mass and strength, as well as the ability to alleviate emotional and cognitive deficits in the aging population (Tsai et al., 2021). Furthermore, formulation that included *Lacticaseibacillus paracasei* PS23 together with omega-3 polyunsaturated fatty acids, and leucine, was able to counteract the progression of sarcopenia and sarcopenic-defining parameters in older adults (Rondanelli et al., 2022).

## 5 Pharmacological and non-pharmacological interventions

### 5.1 Pharmacological interventions

Aging is a complex process influenced by diverse biological mechanisms, which collectively contribute to the deterioration of overall health and increased susceptibility to age-related diseases. Pharmacological treatments involve using drugs and compounds designed to target specific mechanisms associated with age-related diseases. These interventions often focus on cellular and molecular pathways that target the hallmark of aging, such as those related to inflammation, oxidative stress, DNA stability, mitochondrial dysfunction, and cellular senescence (López-Otín et al., 2023). Promising pharmacological candidates include drugs that target various signaling pathways implicated in aging, such as mTOR, AMPK, and Sirtuin1, and compounds that mimic the effects of CR, a dietary approach known to extend lifespan in various organisms (Hassani et al., 2022; Kulkarni et al., 2022).

#### 5.1.1 Rapamycin

Rapamycin, a macrolide compound derived from *Streptomyces hygroscopicus*, has attracted attention not only for its effective immunosuppressive properties but also for its impact on longevity, as it is the first pharmacological compound shown to extend lifespan in mammals (Harrison et al., 2009; Ehninger et al., 2014; Arriola et al., 2016). Rapamycin exerts its anti-proliferative properties by inhibiting the mammalian target of rapamycin (mTOR) pathway, which is a central regulator of cellular growth and it is known for its key role in the aging process. Mechanistically speaking, it acts on inducing autophagy and suppressing protein synthesis (Li et al., 2016). Inhibition of mTOR has been correlated with lifespan extension in mice and other organisms (Lamming et al., 2013). The ability of rapamycin to extend lifespan has prompted extensive research into its potential as an anti-aging intervention. Although the translational implications for human longevity are still under investigation, its promising therapeutic applications are supported by robust preclinical data (Kulkarni et al., 2022).

#### 5.1.2 Metformin

Metformin, a widely prescribed antidiabetic medication known for its broad efficacy in managing glucose metabolism, is emerging as one of the most promising gerotherapeutic drugs due to its potential role in extending lifespan. The primary mechanism of action involves the regulation of cellular energy metabolism through the activation of adenosine monophosphate-activated protein kinase (AMPK) (Cho et al., 2015), reduction in insulin levels (Liu et al., 2022), and modulation of mitochondrial function (Konopka et al., 2019). Metformin’s regulation of protein synthesis in mitochondria, coupled with the activation of AMPK, influences various cellular processes with implications for aging and longevity, including promoting autophagy.

In addition to its pivotal role in glycemic control, metformin has been associated with beneficial effects on diverse health outcomes. Clinical and observational studies have linked metformin to positive impacts on cardiovascular disease, Alzheimer’s disease in diabetic patients (Novelle et al., 2016; Campbell et al., 2017; Mohammed et al., 2021), and reduced mortality (Campbell et al., 2018).

The TAME (Targeting Aging with MEtformin) study is a clinical trial exploring metformin’s potential as an intervention to address aging and age-related diseases, testing the concept of “geroprotection.” This novel approach aims to delay the onset of multiple age-related diseases and extend the health span. Involving a diverse group of participants aged 65 to 79 with or at risk for age-related diseases, the study utilizes a randomized, double-blind, placebo-controlled design to rigorously evaluate metformin’s geroprotective effects (Barzilai et al., 2016).

#### 5.1.3 Caloric restriction mimetics

CR - as mentioned previously in this review, has long been associated with enhanced longevity and delayed aging across various organisms, primates and mice included (Fontana et al., 2010; Mattison et al., 2017; Xie et al., 2022). However, the same degree of beneficial effects has not been observed in humans. Additionally, the metabolic and cellular adaptations induced by CR pose challenges for its practical application in everyday life. In response, the concept of caloric restriction mimetics (CRMs) has emerged, aiming to replicate the beneficial effects of CR without necessitating stringent dietary regimens.

CRMs are widely present in food and function by targeting key cellular pathways implicated in the response to reduced caloric intake. This includes the activation of sirtuins, AMPK, and mTOR pathways, all of which play crucial roles in cellular metabolism, energy sensing, and stress response (Madeo et al., 2019).

Several compounds have garnered attention as potential CRMs; Resveratrol, spermidine, aspirin, quercetin, curcumin, and nicotinamide are among the most investigated molecules. Despite the optimism surrounding these compounds, challenges persist, including concerns about safety, specificity, and efficacy in humans, as they have been tested in small-case studies (Hofer et al., 2022; Hofer et al., 2022).

#### 5.1.4 Anti-inflammatory treatments

Systemic chronic inflammation, often referred to as ‘inflammaging,’ is known to exacerbate the hallmarks of aging and to significantly contribute to the progression of age-related diseases (Li Z. et al., 2023). This ongoing inflammatory state is the result from the accumulation of cellular damage, immune system dysregulation, and the presence of pro-inflammatory factors over time (López-Otín et al., 2023).

Managing inflammaging is an attractive target for improving health in old age. In addition to the aforementioned caloric restriction (CR) mimetics, senolytics have shown promise in reducing age-related conditions such as sarcopenia, cataracts, vascular pathology, and cognitive decline in animal models (Baker et al., 2011; Roos et al., 2016; Ogrodnik et al., 2021), such as dasatinib and quercetin, work by eliminating senescent cells, which reduces the production of pro-inflammatory cytokines including TNFα, IL-6, and IL-8, as well as alleviating activation of signaling pathways such as NF-κB and JAK/STAT3—key contributors to chronic inflammation (Dugan et al., 2023).

Despite these promising results, questions remain about the duration of benefits and potential adverse effects of long-term senolytic use, which must be addressed through clinical trials before broad implementation.

### 5.2 Non-pharmacological interventions

The evolving field of longevity medicine seeks to provide individuals with extended years of life and, crucially, an improved quality of life throughout their extended years. While pharmaceutical advancements continue to drive medical progress, non-pharmacological interventions offer a distinctive and compelling approach, capitalizing on the potential of holistic health and wellbeing. Most known and evidence-based non-pharmacological interventions include sauna bathing, cold water immersion, cryotherapy, intermittent hyperoxia-hypoxia training, hyperbaric oxygen chamber, and different intravenous therapies.

#### 5.2.1 Sauna

There is large evidence data indicating that sauna bathing has reduces the risk of cardiovascular diseases (CVD), neurodegenerative diseases, and pulmonary diseases, as well as pain relief properties (Kunutsor et al., 2018a; Ahokas et al., 2023). Sauna is form of passive thermal therapy by exposure to high environmental temperature from 80°C to 100°C for a brief period, ranging from 5–20 min depending on the individual needs (Kukkonen-Harjula and Kauppinen, 2006). A number of studies have assessed the effect of sauna over cardiovascular conditions, including hypertension, ischemic stroke and CVD mortality (Zaccardi et al., 2017; Kunutsor et al., 2018b; 2018a). A few longitudinal studies assessed sauna bating and CVD mortality over 24 years, concluding a 47% risk reduction for hypertension and 62% risk reduction for stroke in individuals who practiced frequent sauna baths (Zaccardi et al., 2017; Kunutsor et al., 2018b). Additionally, sauna bathing has been associated with 65% reduced risk of neurodegenerative diseases such as Alzheimer’s and dementia (Laukkanen et al., 2017). Application of local heat therapy and sauna is shown to promote muscular vascularity and improve muscle soreness (Heinonen et al., 2011; Ahokas et al., 2023). The sauna has a pleiotropic effect that has been shown to increase high-density lipoprotein cholesterol (HDL-C), decrease low density lipoprotein cholesterol (LDL-C), to have antioxidant effect and reduce reactive oxygen species, improves endothelial function and immune support (Laukkanen et al., 2018).

Alternative method to sauna therapy is infrared sauna that uses infrared heaters to emit infrared light that is absorbed by the skin and underlying tissues, creating heat within the body. The heat raises the core temperature, inducing a state of therapeutic warmth and sweating response. Typically, infrared sauna has lower temperature and lower level of humidity. Infrared sauna has a pain-relieving properties that help treat muscle soreness, stiffness and chronic fatigue (Oosterveld et al., 2009; Ahokas et al., 2023).

#### 5.2.2 Cold immersion therapy

Intermittent changes of sauna bathing and cooling with cold water immersion therapy is beneficial with regards to cardiorespiratory fitness, endocrine systema and thermoregulation (Podstawski et al., 2021; Søberg et al., 2021). Cold water immersion consists of body exposure to cold water between 0°C and 10°C for a short period of time. Alternative method is cryotherapy chambers, where the cold exposure happens in a controlled cold environment using liquid nitrogen or electric cooling system. Cryo chambers create much lower temperature up to −129°C. Brief immersion in cold water elicits cold shock response, activation of the sympathetic nervous system and improved thermogenesis (Søberg et al., 2021). There has been some adverse effect associated with drastic temperature changes such as ventricular and atrial arrythmia in some individuals with CVD (Schmid et al., 2009). The acute thermodynamic stress that the body endures during hot and cold therapy, stimulates the immune function, and improves innate immunity (Brazaitis et al., 2014). While the effect of hot and cold therapy is known for optimizing physical health, there are also evidences supporting the beneficial effect of cod water immersion or cryo chambers on cognitive health, improved focus, mood enhancement and depression (Miller et al., 2011; Rymaszewska et al., 2021; Kelly and Bird, 2022).

#### 5.2.3 (Infra) red light therapy

Red light therapy, also known as photo biomodulation or low-level laser therapy, involves the use of red or near-infrared light to stimulate various cellular processes in the body (de Freitas and Hamblin, 2016). A proposed mechanism of action is that the photons dissociate inhibitory nitric oxide from the enzyme, leading to an increase in electron transport, mitochondrial membrane potential and ATP production. That leads to activation of transcription factors related to protein synthesis, and cell proliferation (de Freitas and Hamblin, 2016). Red light therapy in different wavelengths is used clinically in several directions, most successfully to treat skin disorders such as scar tissue, acne, and wrinkles (Nam et al., 2017; Santiago et al., 2022). Near-infrared light therapy increases collagen production in the skin that improves the firmness and elasticity of the skin layers and reduce inflammation and redness (Nam et al., 2017). Additional therapeutic benefits of longer wave lengths of red light have been investigating with regards to muscle recovery, joint pain relief and local inflammation (Wickenheisser et al., 2019; Ailioaie and Litscher, 2021; González-Muñoz et al., 2023).

#### 5.2.4 Intermittent hyperoxia-hypoxia training (IHHT)

IHHT training involves alternating cycles of adaptation in normobaric hypoxia and then reoxygenation and hyperoxia. It is achieved by breathing hypoxic gas mixture through a mask in a controlled environment for few minutes in each cycle. The adaptation of episodes of hypoxia and hyperoxia is a powerful stress factor that triggers physiological reaction in the body and tissues, which improves the overall resilience of the organism (Rybnikova et al., 2020). IHHT enhances cardiac muscle resistance to hypoxia and improves myocardial function (Glazachev et al., 2017). Additionally, IHHT can affect metabolic health as it affect glucose metabolism, by improving glucose tolerance as well as symptoms of obesity and diabetes (De Groote et al., 2018). Similarly, IHHT has been shown to improve lipid profile in patients with already diagnosed metabolic syndrome, as well as improve their anti-inflammatory status (Afina et al., 2021). IHHT also has a direct effect on cognitive function and cerebral vascular health, due to improved hypoxia tolerance and improved protection of mitochondrial damage (Li et al., 2016; Rybnikova et al., 2022). Moreover, patients with already diagnosed mild cognitive impairment and Alzheimer’s diseases show improved cognitive test scores after IHHT (Serebrovska et al., 2019). Recent findings associate IHHT with telomere stabilization and protection form cellular senescence and apoptosis, which has a beneficial effect on age-related diseases (Tessema et al., 2022).

#### 5.2.5 Hyperbaric oxygen therapy (HBOT)

HBOT uses the power of 100% oxygen in environmental pressure higher than one absolute atmospheres (ATA) to promote healing and enhance overall wellbeing. A typical HBOT continue for approximately 90 min with some changes of ATA and breaks with normal air in between. There have been numerous clinical indications for using HBOT including for venous embolism, severe carbon monoxide poisoning, anemia, chronic osteomyelitis, and idiopathic sensorineural hearing loss (Kirby et al., 2019). Along with the obvious benefit of delivering more oxygen to the tissues, HBOT has beneficial effect on vascular health, hemoglobinopathies, improved circulation (Lindholm and Lundgren, 2009). HBOT has also been implicated as a great tool for improving metabolic health and glucose regulation by improving peripheral insulin sensitivity (Wilkinson et al., 2012). For the first time in humans, recent findings have shown that HBOT can modulate the pathophysiology of skin aging, by angiogenesis and facilitating senescent cells clearance (Hachmo et al., 2021). A randomized clinical trial demonstrated that HBOT induces cognitive enhancement, improved attention, processing speed and executive function in aging adults, causing regional changes in cerebral blood flow (Hadanny et al., 2020). Most promising evidence regarding HBOT is its significant effect on increasing telomere length and clearance of senescent cells in aging population (Hachmo et al., 2020).

#### 5.2.6 Intravenous (IV) therapy

IV therapy is an innovative solution to directly infuse the body with carefully curated blend of essential nutrients, vitamins, mineral and health-promoting compounds. By delivering the nutrients directly to the blood stream and bypassing the digestive tract allows quicker absorption. A recent Mendelian randomized study found that some antioxidants (β-carotene, lycopene and uric acid), vitamins (A, B6, B12, C, D and K1), and minerals (Ca, Mg, Cu) are causally related to the development of metabolic syndrome (Li and Song, 2023). IV therapies can compensate for these well-known relationships between deficient levels of micronutrients and age-related conditions. Typical IV therapy includes some amino acids, vitamin C, vitamins from group B, vitamin D, some minerals, nutraceuticals such as glutathione. Prolonged infusion of glutathione has been indicated to improve myocardial cells survival (Tanzilli et al., 2019). A personalized mix of different nutrients can be used to increase energy levels, improve the immune system, enhanced skin health.

## 6 Experimental strategies

At the top of the Longevity Pyramid are the experimental strategies (Figure 1; Table 3), including gene editing, mRNA-based therapies, stem cells, and extracellular vesicles therapies, along with advancements in tissue engineering, which represent promising solutions for targeting the hallmarks of aging and reshaping the landscape of aging-related research and interventions (Mishra et al., 2022).

**TABLE 3:** Examples of experimental strategies in aging-related research, which include gene editing technologies, mRNA-based therapies, stem cells (SCs) and extracellular vesicles (EVs) therapies and tissue engineering strategies. The table is not meant to be exhaustive, but rather to provide an illustrative overview of some of the primary applications of these strategies.

	Experimental strategy	Functional target	Molecular target (model system)	References
Gene editing	Recombinant vectors	Telomere attrition Mitochondrial dysfunction	*TERT* gene (mice)	Bernardes de Jesus et al. (2012), Bar et al. (2014)
	CRISPR-Cas9	Telomere attrition Mitochondrial dysfunction	*TERT/*telomerase (mice) mtDNA (human cell lines)	Dai et al. (2019) Jo et al. (2015)
mRNA-based therapies	mRNA encoding TERT	Telomere attrition	*TERT* gene (human cell lines)	Ramunas et al. (2015)
Stem Cell therapies	Intravenous allo-hMSCs	Aging frailty	Inflammatory markers (humans)	Tompkins and Landin (2017)
Extracellular vesicles (EVs) therapies	Intravenous transplantation	Premature Ovarian Failure	PI3K/AKT signaling pathway (mice)	Li et al. (2023a)
	ADSC-sEVs injection	Telomere attrition Oxidative stress Cellular senescence	Telomere length Lipid peroxidation, Protein carbonylation Lamin B1 (mice)	Sanz-Ros et al. (2022)
Tissue engineering	Hydrogels	Cardiac tissue	hiPSC-CMs (3D human cardiac tissue)	Lee et al. (2017)
	Microfabrication	Cardiac tissue	Rat ventricular Cardiomyocytes (3D cardiac patch)	Feiner et al. (2016)
	Bioprinting	Cardiac tissue	Human cardiomyocytes (3D-bioprint collagen)	Lee et al. (2019)
	Natural scaffold	Cartilage	TGF-β3, DCB, ECM (rats)	Yang et al. (2021)
	Subcutaneous implantation of PEC cells containing device	Type-1 diabetes	PSCs pancreatic endoderm cells (human cells)	Shapiro et al. (2021)

Abbreviations: ADSC-sEVs, White adipose tissue-derived small extracellular vesicles; PEC, cells–photoelectrochemical cells; TERT, Telomerase Reverse Transcriptase; PI3K–phosphatidylinositol 3-kinase; AKT, serine/threonine protein kinase; hiPSC-CMs, Human induced pluripotent stem cell-derived cardiomyocytes; TGF, Transforming growth factor; DCB, drug-coated balloon; ECM, extracellular matrix.

Gene editing technologies, such as CRISPR-Cas9, enable precise modification of the genome, offering large opportunities to target age-related genes and pathways (Yu et al., 2023). mRNA-based therapies provide a versatile tool for fine-tuning gene expression (Qin et al., 2022). Stem cells (SCs), with their regenerative capabilities, hold the potential to replenish and rejuvenate aging tissues. In particular, stem cell intravenous treatment (IV), in which SCs are directly infused into the patient’s bloodstream, is gaining growing attention as a promising option to promote tissue repair (Watt and Driskell, 2010; Garay, 2023). Extracellular vesicles (EVs), small vesicles deriving from multiple stem cells with an intricate cell-to-cell communication ability, offer novel solutions for intercellular signaling and therapeutic delivery (Barile and Vassalli, 2017). Tissue engineering strategies, combining biomaterials and cellular components, open new horizons for the creation of functional and regenerative tissues (Khademhosseini and Langer, 2016). Examples of applications of tissue engineering are heart regenerative therapies (Tenreiro et al., 2021). Examples of experimental strategies in aging-related research are shown in Table 3.

## 7 Discussion

The “*Longevity Pyramid*” concept, as elaborated in this review, illustrates the progressive intervention levels in longevity medicine, emphasizing the importance of a multi-layered approach. This model encourages the synthesis of diagnostic and preventive measures, lifestyle modifications, and advanced therapeutic interventions. The role of diagnostics in early detection and the utilization of biomarkers and genetic/epigenetic testing is pivotal. The integration of wearable healthcare technologies and physiological measurements underlines the transition towards a more data-driven, personalized approach in healthcare. Lifestyle choices, including exercise, diet, and oral health, are critical in influencing the aging process. The impact of physical activity, particularly resistance training, on various physiological systems emphasizes the holistic nature of these interventions. Dietary patterns like caloric restriction, intermediate fasting, plant-based diets, and the Mediterranean diet demonstrate the significant influence of nutrition on longevity. The exploration of various supplements and pharmacological agents like NAD + precursors, spermidine, and metformin highlight the growing interest in targeting specific aging pathways and hallmarks. These approaches, while promising, require further validation in long-term human studies. Non-pharmacological interventions such as sauna therapy and hyperbaric oxygen therapy, along with experimental strategies involving gene editing and stem cell therapies, present innovative frontiers in longevity medicine. However, these require cautious optimism and rigorous validation.

## 8 Limitations and future directions

Despite the potential breakthroughs in longevity medicine, several significant limitations should be considered. A primary limitation is the lack of robust, long-term data on many of the interventions discussed. While some dietary approaches and lifestyle interventions have shown promise in observational studies, randomized controlled trials (RCTs) that evaluate the effects of these interventions over extended periods remain sparse (Sebastiani et al., 2017). For example, while caloric restriction and intermittent fasting have been shown to improve biomarkers of aging in short-term studies, long-term adherence and the potential risks associated with nutrient deficiencies are not fully understood (Longo et al., 2021). Additionally, human studies on caloric restriction mimetics and other supplements such as NAD + precursors, spermidine, and metformin are still in the early stages. Although promising in preclinical studies, these interventions require long-term, large-scale RCTs to establish efficacy and safety in humans. Without these trials, the practical application of these strategies remains limited, and their long-term effects on healthspan and lifespan are uncertain (Longo et al., 2021).

Another limitation is the variability in individual responses to aging interventions. Factors such as genetic predisposition, baseline health, lifestyle choices, and environmental influences significantly affect how individuals respond to longevity interventions (De Magalhães, 2014). Personalized medicine, while a growing field, is still not fully capable of predicting these individual outcomes with accuracy. The emerging field of biomarker analysis and the use of epigenetic clocks offer some hope in this area, but the accuracy of these tools in predicting both biological age and individual responses to interventions is still under scrutiny (Moqri et al., 2023). Additionally, wearable technologies that offer continuous monitoring of health parameters show potential for individualized health strategies (Paolillo et al., 2022), but their widespread use raises issues related to data privacy, accessibility, and the reliability of the data collected over time.

The translation of findings from animal models to humans also remains a significant limitation (Leenaars et al., 2019). Many of the pharmacological interventions and experimental strategies, such as gene editing and stem cell therapies, have shown remarkable effects in extending lifespan and reducing age-related decline in animal models (Mitchell et al., 2015). However, the complexity of human aging, coupled with ethical considerations, makes the direct application of these strategies in humans challenging. For instance, gene editing technologies like CRISPR-Cas9 present exciting possibilities for addressing age-related diseases, but concerns regarding off-target effects, long-term safety, and the ethical implications of genetic modifications need to be thoroughly addressed before such interventions can become mainstream (Caobi et al., 2020).

Moreover, many of the discussed interventions are currently only accessible to select groups of people, either due to high costs, lack of regulatory approval, or geographical disparities in healthcare infrastructure (Scott et al., 2021). This raises ethical concerns about equity in healthcare, as life-extending therapies may be limited to those with the financial means to afford them. The societal impact of extending human lifespan also needs to be critically examined, including the implications for population growth, resource allocation, and healthcare systems (Scott et al., 2021). Without careful consideration of these issues, the development and implementation of longevity interventions could exacerbate existing health inequalities.

## 9 Future research needs

Looking forward, future research should prioritize long-term, multicenter clinical trials that investigate the effects of combined lifestyle, dietary, and pharmacological interventions on both healthspan and lifespan. In particular, there is a need for studies that examine the interactions between different interventions, such as the combined effects of exercise, diet, and pharmaceutical agents like metformin or rapamycin, to provide a more holistic understanding of how these factors work together to promote longevity. Additionally, there is a pressing need to develop better biomarkers for aging that are not only predictive but also actionable, allowing for more targeted interventions tailored to an individual’s specific biological age and health profile.

Research should also explore the integration of artificial intelligence (AI) and machine learning to enhance the precision of personalized medicine (Zhavoronkov et al., 2019). AI can play a critical role in processing vast amounts of health data, identifying patterns, and predicting outcomes, ultimately helping to tailor interventions based on real-time data from wearable devices and biological markers. Future studies should focus on the development of AI-driven health platforms that combine genetic, epigenetic, and environmental data to optimize intervention strategies for individuals.

Moreover, the future of longevity medicine will likely require interdisciplinary collaborations that involve experts in genetics, bioinformatics, ethics, healthcare policy, and clinical practice. These collaborations will be essential for addressing the ethical, legal, and social implications of emerging technologies, such as gene editing and stem cell therapies, in the context of human longevity. Regulatory frameworks will also need to evolve to accommodate the rapid advancements in this field, ensuring that safety and efficacy standards are met while enabling innovation.

## 10 Next steps in longevity medicine

The next steps in longevity research should also include addressing the practical challenges of integrating longevity medicine into mainstream healthcare. Developing cost-effective interventions that can be scaled across diverse populations is critical to ensuring that longevity advancements benefit all segments of society. Policies aimed at reducing healthcare disparities and making cutting-edge treatments more accessible will be essential in creating a fairer healthcare landscape. This is especially important as the field of longevity medicine moves from experimental to practical application.

Ultimately, the future of longevity research will depend on a balanced approach that combines scientific innovation with ethical responsibility. The excitement surrounding the potential for extended healthspan and lifespan must be tempered with caution, ensuring that interventions are both safe and accessible. As longevity medicine continues to evolve, it will be crucial to maintain a focus on improving quality of life, not merely extending the number of years lived.

The review findings point towards the need for tailored healthcare interventions, considering individual genetic, lifestyle, and environmental factors. Shifting from a reactive to a preventive healthcare model is crucial. Early intervention strategies could significantly alter the trajectory of aging and related diseases. The interplay between various levels of the Longevity Pyramid underscores the necessity for an integrated approach, combining lifestyle, medical, and technological strategies. The advancements in longevity medicine bring forth ethical and societal implications, particularly in resource allocation, healthcare accessibility, and the societal impact of extending human lifespan.

## 11 Conclusion

This comprehensive review underscores the multifaceted nature of longevity medicine. By outlining a structured approach through the Longevity Pyramid, it highlights the significance of integrating (i) early diagnostics, (ii) preventive strategies, (iii) lifestyle modifications, (iv) and advanced therapeutic interventions to promote healthy aging and extend lifespan. Future research should focus on refining these strategies, ensuring their practical applicability, and understanding their long-term effects on human health and society. The rapid advancements in this field, coupled with growing global interest in aging, present an exciting, yet challenging, Frontier in healthcare, necessitating a balanced approach that considers both individual wellbeing and broader societal implications. The journey up the Longevity Pyramid is not a solitary climb but a collective endeavor, requiring collaboration across various disciplines and stakeholders. As we continue to ascend, it is imperative to remain guided by evidence-based practices, ethical considerations, and a commitment to improving the quality of life across the lifespan.
